# Supplementary material for: k-dependent modulation of intrinsic spin-orbit interaction in MoSe2 induced by proximity to amorphous Pb
Source: Nat Commun. 2025 Dec 24;17:1076. doi: 10.1038/s41467-025-67833-y (PMC12852670; doi:10.1038/s41467-025-67833-y)
Supplement: Supplementary file 1 — Supplementary Information [file 41467_2025_67833_MOESM1_ESM.pdf]

k-dependent modulation of intrinsic spin-orbit interaction in $MoSe_2$ induced by proximity to amorphous Pb

(Supporting information)

Fatima Alarab [1], *Ján Minár* [2], *Procopios Constantinou* [1], *Dhani Nafday*[3], *Aki Pulkkinen*[2], *Thorsten Schmitt* [1], *Xiaoqiang Wang* [1] *and Vladimir N. Strocov* [1]

[1]Swiss Light Source, Paul Scherrer Institute, 5232 Villigen-PSI, Switzerland
[2]New Technologies Research Centre, University of West Bohemia, 301 00 Plzeň, Czech Republic
[3]Asia Pacific Center for Theoretical Physics, 37673 Pohang, Gyeongbuk, South Korea

1. ARPES data on $MoSe_2$ through the series of Pb depositions

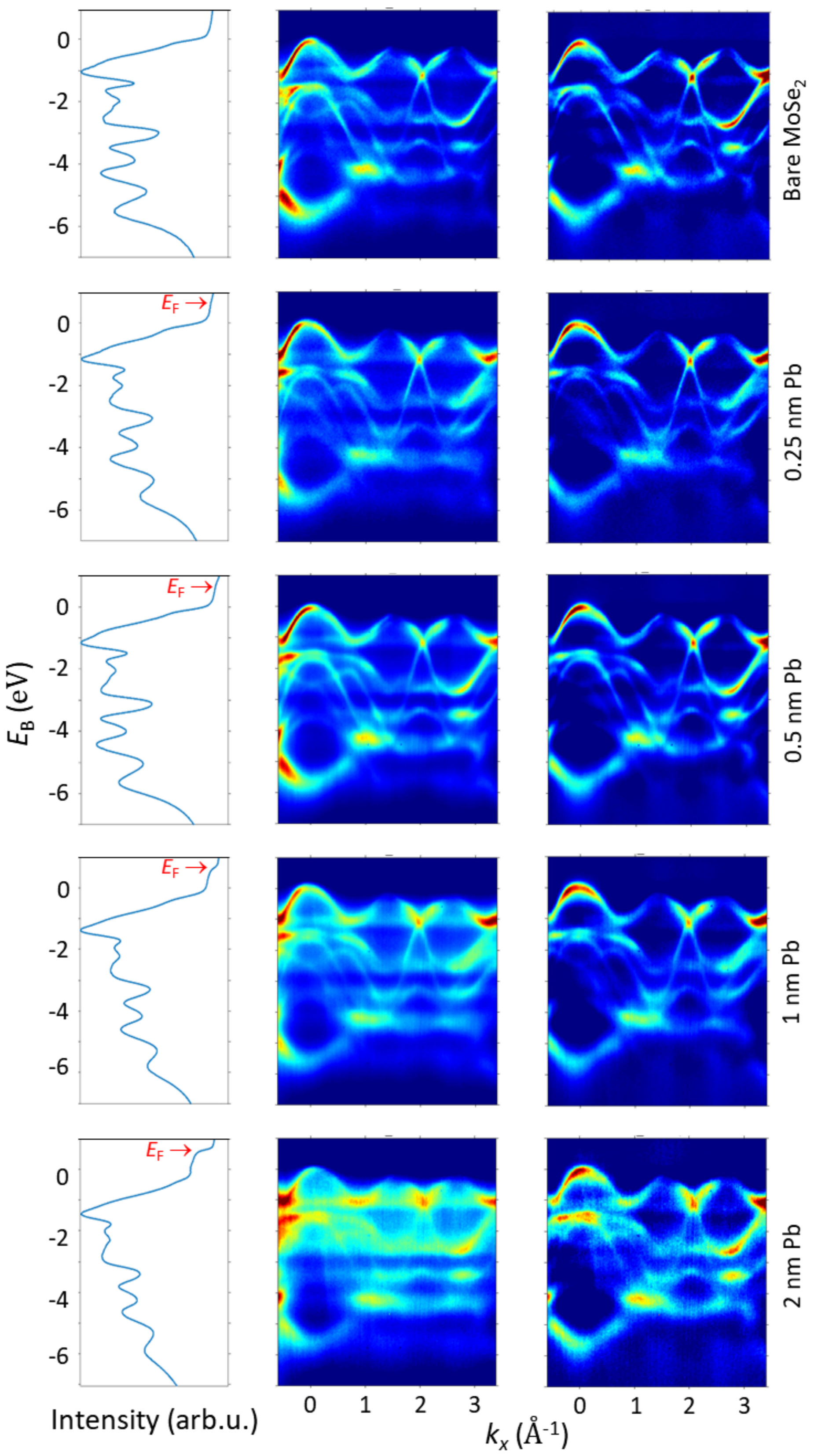

Fig. SI1-1. ARPES intensity along Γ_1KMK ($h\nu$=737 eV) through the whole series of Pb depositions: (*left column*) angle-integrated, including the incoherent background, (*central*) raw images, and (*right*) raw images with the angle-integrated subtracted. The incoherent background from the amorphous Pb overlayer progressively increases with its thickness. Besides this increase as well as broadening of the spectral peaks and their slight **k**-dependent energy shifts, the Pb overlayer does not critically affect the $MoSe_2$ electronic states.

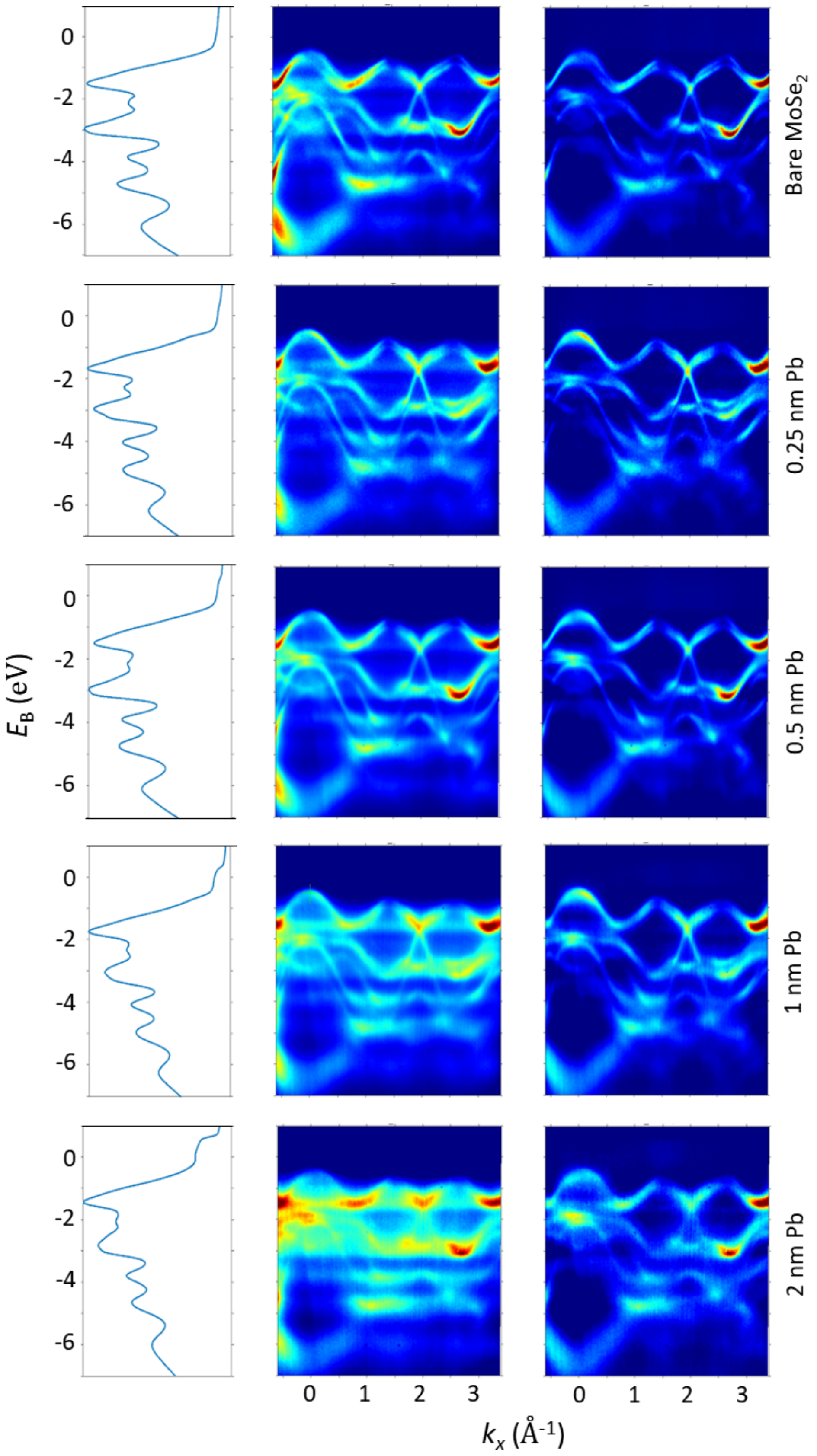

Fig. SI1-2. ARPES data along AHLH (*hv* = 715 eV) represented similarly to Fig. SI1-1.

2. ARPES spectral background through the Pb-deposition series

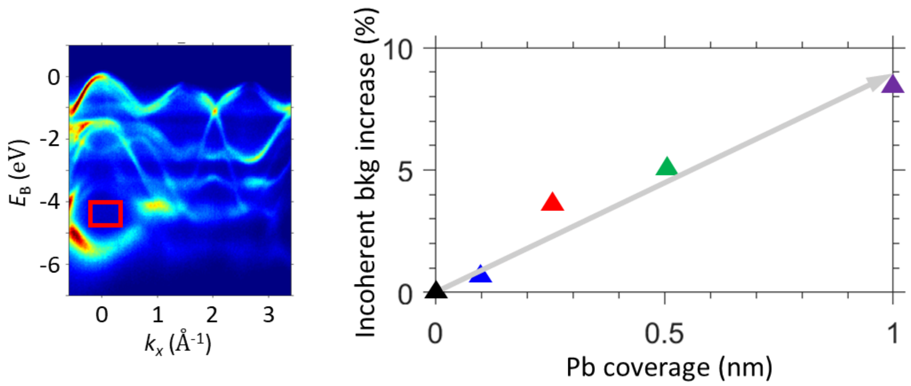

Fig. SI2. (*left*) ARPES image along Γ_1KMK (*hv*=737 eV) where the red rectangle marks the region away from the band диспersions where the spectral background is measured; (*right*) the background dependence on the Pb deposition.

3. ARPES data through the whole series of Pb depositions

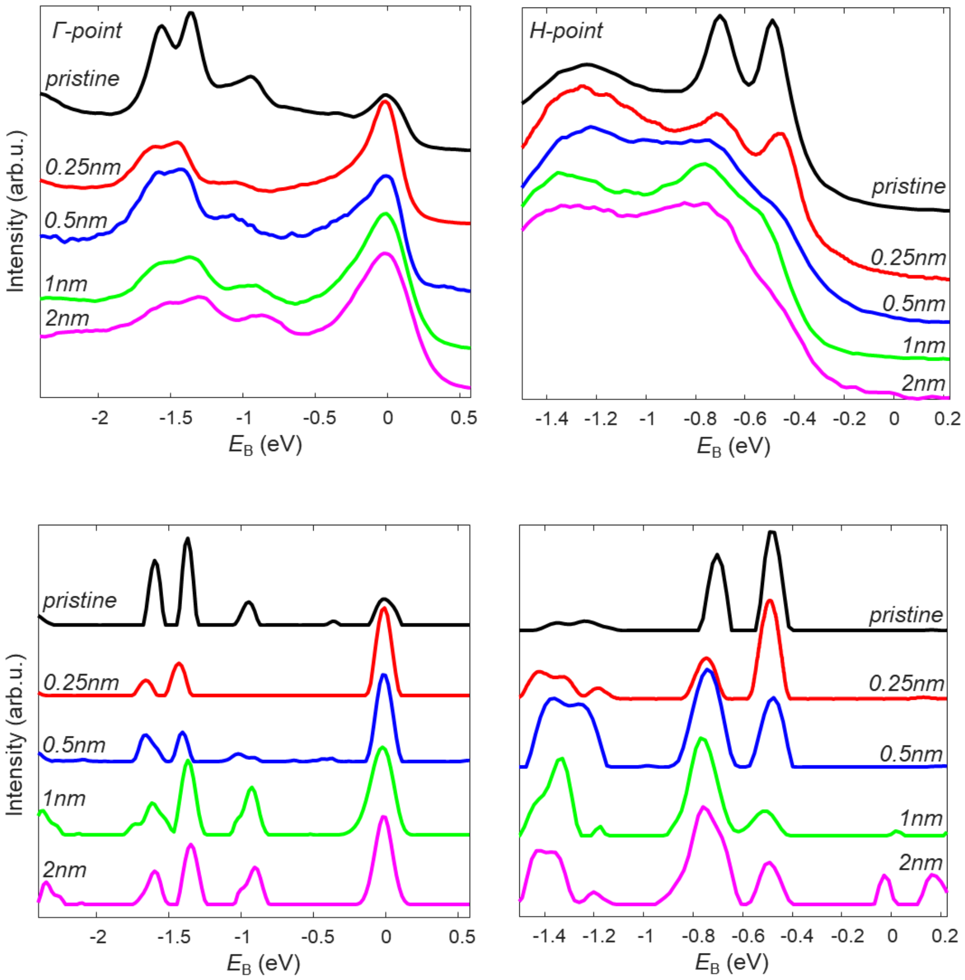

Fig. SI3: ARPES data in the Γ_1-point (hv = 737 eV) and H-point (hv = 715 eV) through through the whole series of Pb depositions. The data are represented as (*top panels*) the EDCs and (*bottom*) the $-d^2I/dE^2 > 0$ plots.

4. Effect of electric field on $MoSe_2$ band structure

To quantify the effect of an electric field on the band structure, we have performed DFT-based calculations with the full potential linearized augmented plane wave (FP-LAPW) code ELK with a static out-of-plane electric field applied to the $MoSe_2$ layers and having the periodicity of the unit cell. The maximal field strength was 1 V/nm, a safe upper estimate for the field that could potentially be induced by the Pb overlayer. The potential created by this field lifts the spin degeneracy of the bands, classified by their out-of-plane (largest) spin component S_z, which now become doublets (blue and red pairs in Fig. SI3-1). The energy separation of the bands Δ_{bs} is evaluated in this case as the difference of the central energies of the upper and lower doublets (marked by green lines in the K- and H-points).

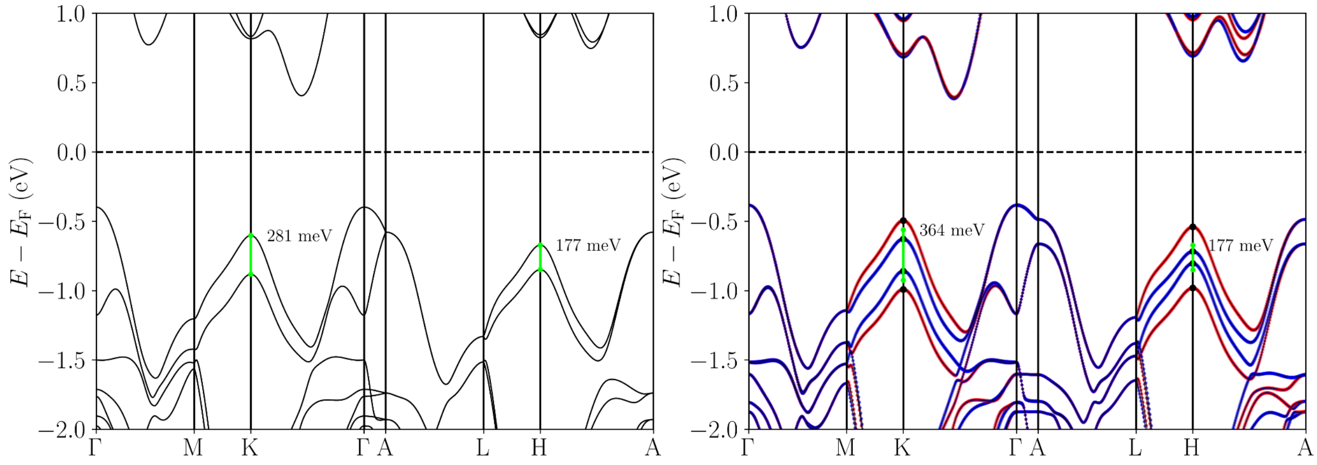

Fig. SI4-1: Band structure of $MoSe_2$ without (*left*) and with (*right*) an out-of-plane electric field of 1 V/nm. The red and blue colours in the right panel indicate the out-of-plane spin character of the bands. The band splittings at the K and H-points between the centers of spin-split band doublets are marked by green dashes. The energy scale is relative to the Fermi level. It is worth noting in passing that the 1.0 V/nm electric field reduces the fundamental band gap by 37 meV.

Our calculations in Fig. SI3-1 show that at the K-point, where the separation is dominated by the interlayer interaction, the splitting increases from 281 to 364 meV at E = 1.0 V/nm. At the same time, the separation at the H-hotspot, attributed exclusively to SOI, stays at 177 meV with only a μeV-scale variation. These splittings as a function of the electric field strength are presented in Fig. SI3-2. Also, the calculations deny any effect of the field in the Γ_1-hotspot. These theoretical results fail to reproduce even quantitatively the experimental data where, upon deposition of Pb, no splitting of the bulk bands (or their additional broadening, if the splitting is below the experimental resolution and lifetime energy broadening) is observed in the K-point and an increase of Δ_{bs} by ~60 meV and ~30 meV is observed in the H- and Γ_1-hotspots, respectively. This fact denies any involvement of the field-induced Rashba-type SOI in the experimentally observed increase of Δ_{bs} in the H- and Γ_1-hotspots. Our analysis goes along with the theoretical study on twisted graphene/TMDC interfaces [21] where the electric-field dependence modulates the Rashba SOI but does not affect the intrinsic valley-Zeeman one.

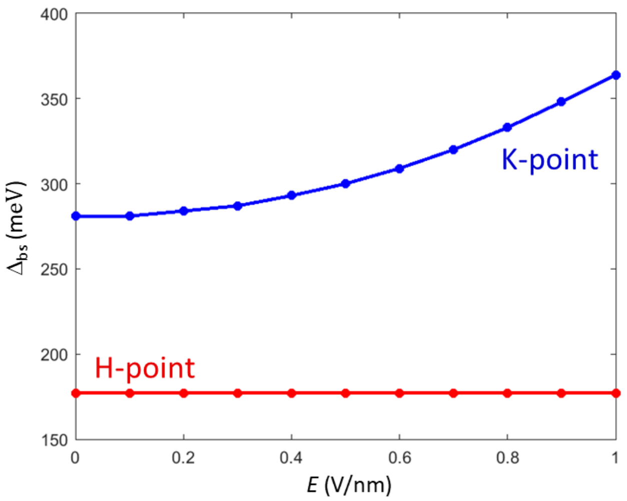

Fig. SI4-2: The band splittings at the K- and H-points calculated as the difference between the centers of the upper and lower spin-split band doublets (green dashes in Fig. SI3-1) as a function of the electric-field strengths.

5. Al deposition on $MoSe_2$: Band splitting in the SO hotspots

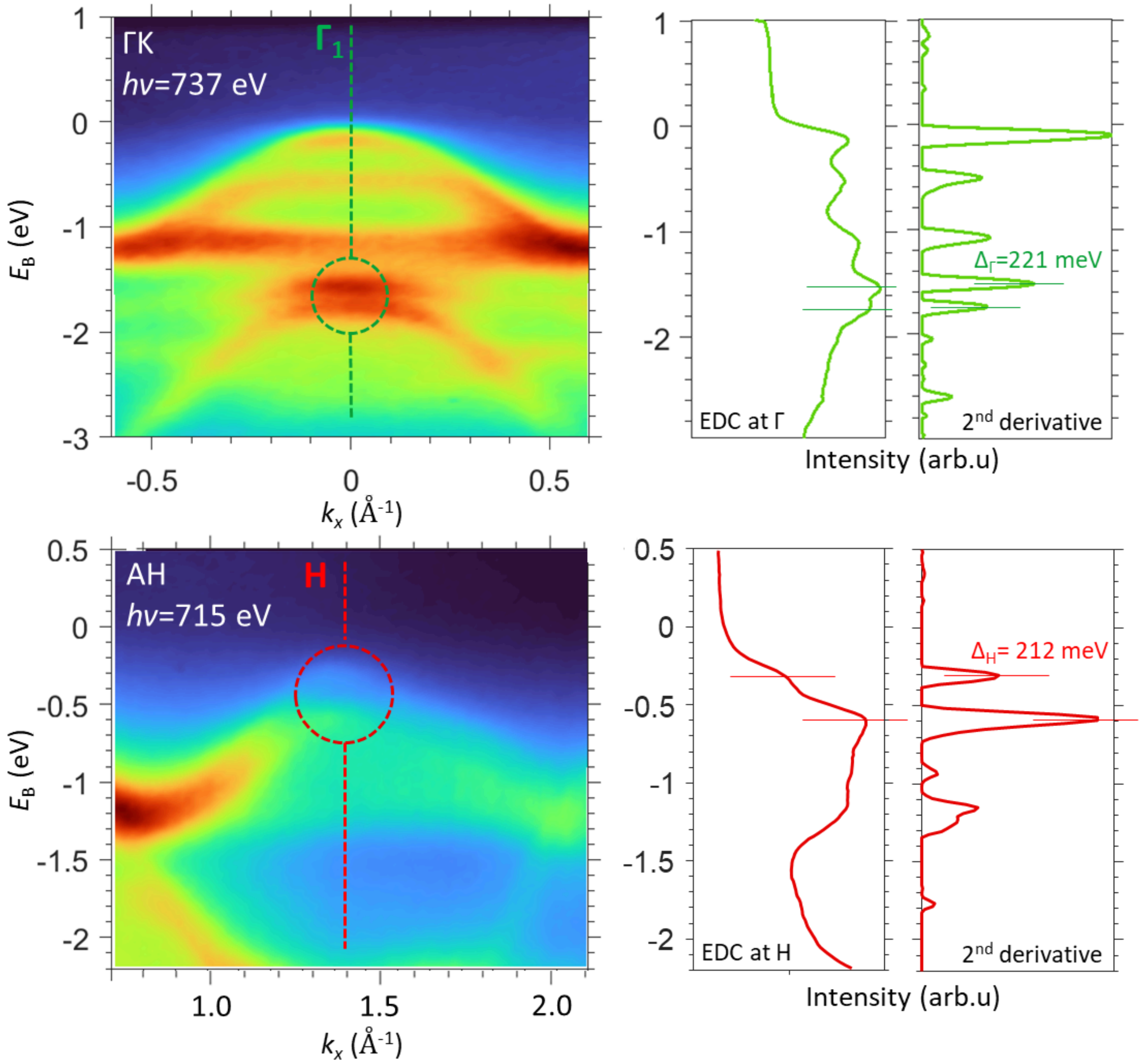

Fig. SI5: ARPES images around the Γ_1- and H-hotspots with the EDC and $-d^2I/dE^2 > 0$ plots in these points after the deposition of 0.25 nm of Al. In contrast to Pb, the Al overlayer does not change Δ_{bs} within the experimental accuracy ±3 meV.

6. Reproducibility of ARPES data through $MoSe_2$ samples

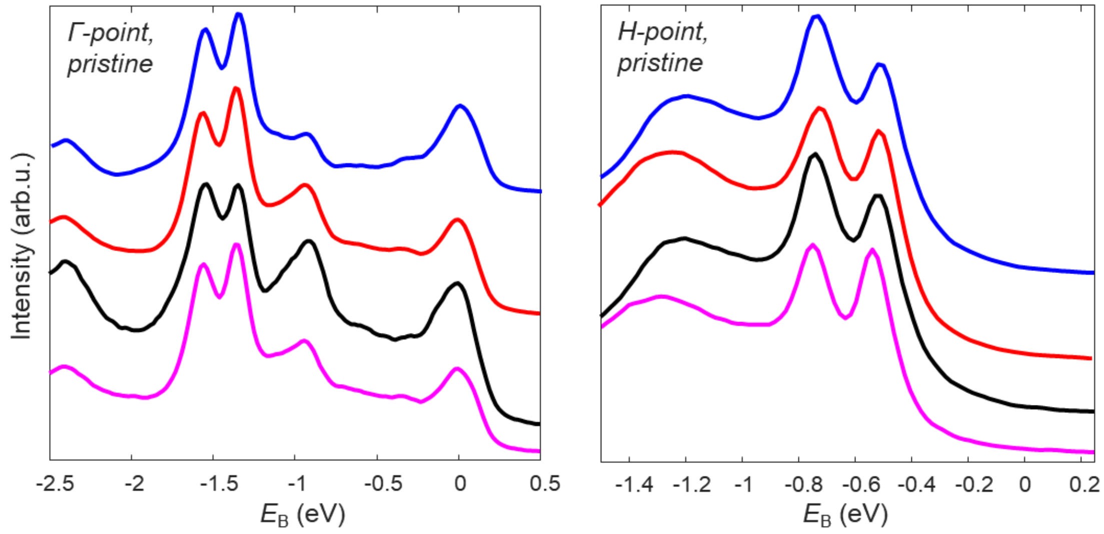

Fig. SI6. EDCs of the ARPES intensity in the Γ_1-point ($h\nu$=737 eV) and H-point ($h\nu$ = 715 eV) through a series of pristine $MoSe_2$ samples. The observed minor difference of the EDCs is of the same order as their variation along each sample.
